# Increased Oxidative Stress and Imbalance in Antioxidant Enzymes in the Brains of Alloxan-Induced Diabetic Rats

**DOI:** 10.1155/2012/302682

**Published:** 2012-05-08

**Authors:** Luciane B. Ceretta, Gislaine Z. Réus, Helena M. Abelaira, Karine F. Ribeiro, Giovanni Zappellini, Francine F. Felisbino, Amanda V. Steckert, Felipe Dal-Pizzol, João Quevedo

**Affiliations:** ^1^Laboratório de Neurociências and Instituto Nacional de Ciência e Tecnologia Translacional em Medicina (INCT-TM), Programa de Pós-Graduação em Ciências da Saúde, Unidade Acadêmica de Ciências da Saúde, Universidade do Extremo Sul Catarinense, 88806-000 Criciúma, SC, Brazil; ^2^Laboratório de Fisiopatologia Experimental and Instituto Nacional de Ciência e Tecnologia Translacional em Medicina (INCT-TM), Programa de Pós-Graduação em Ciências da Saúde, Unidade Acadêmica de Ciências da Saúde, Universidade do Extremo Sul Catarinense, 88806-000 Criciúma, SC, Brazil

## Abstract

Diabetes Mellitus (DM) is associated with pathological changes in the central nervous system (SNC) as well as alterations in oxidative stress. Thus, the main objective of this study was to evaluate the effects of the animal model of diabetes induced by alloxan on memory and oxidative stress. Diabetes was induced in Wistar rats by using a single injection of alloxan (150 mg/kg), and fifteen days after induction, the rats memory was evaluated through the use of the object recognition task. The oxidative stress parameters and the activity of antioxidant enzymes, superoxide dismutase (SOD), and catalase (CAT) were measured in the rat brain. The results showed that diabetic rats did not have alterations in their recognition memory. However, the results did show that diabetic rats had increases in the levels of superoxide in the prefrontal cortex, and in thiobarbituric acid reactive species (TBARS) production in the prefrontal cortex and in the amygdala in submitochondrial particles. Also, there was an increase in protein oxidation in the hippocampus and striatum, and in TBARS oxidation in the striatum and amygdala. The SOD activity was decreased in diabetic rats in the striatum and amygdala. However, the CAT activity was increased in the hippocampus taken from diabetic rats. In conclusion, our findings illustrate that the animal model of diabetes induced by alloxan did not cause alterations in the animals' recognition memory, but it produced oxidants and an imbalance between SOD and CAT activities, which could contribute to the pathophysiology of diabetes.

## 1. Introduction

Diabetes Mellitus (DM) is a heterogeneous metabolic disorder characterized by hyperglycemia [1]. In type 1 diabetes (DM1), which generally develops at a young age (children and early adulthood), the principal defect is an auto-immune-mediated destruction of pancreatic cells, leading to insulin deficiency [2]. In type 2 diabetes (DM2) the principal defect is insulin resistance, leading to a relative insulin deficiency in the liver and peripheral tissues, which leads to overt hyperglycaemia [3]. The hyperglycaemia in turn causes upto a fourfold increase in neuronal glucose, with intracellular glucose metabolism then leads to neuronal damage [4]. In addition to this, the current therapeutic strategies for DM2 are limited [5].

In both the human and animal models, DM is associated with pathological changes in the central nervous system (SNC) that lead to cognitive and affective deficits, and to an increased risk of brain vascular complications [3]. In the animal models of diabetes, several brain alterations have been described, such as increased hippocampal astrocytic reactivity, impaired synaptic plasticity, vascular changes, decreased dendritic complexity, and disturbed neurotransmission [6]. Recently, a significant body of evidence has accumulated to indicate that diabetes has detrimental effects on brain function. A number of investigations have been performed to indicate that memory loss is a consequence of both type I and type II diabetes [7]. Some authors have also reported a reduction in the length and a simplification of the dendritic trees of the hippocampal pyramidal cells in diabetic rodents [6]. There is evidence from the animal models showing that changes in dendritic morphology, probably associated with synaptic disturbances, correlate with alterations in memory and learning abilities [8]. Mitochondria are the principal source of reactive oxygen species (ROS) in cells, as the result of imperfectly coupled electron transport. Oxidative stress is widely accepted as playing a key mediatory role in the development and progression of diabetes and its complications, due to the increased production of free radicals and impaired antioxidant defenses [9]. Several mechanisms can contribute to increased oxidative stress in diabetic patients, especially chronic exposure to hyperglycemia. Accumulated evidence points out that hyperglycemia can lead to elevated ROS and reactive nitrogen species (RNS) production by the mitochondrial respiratory system [10], glucose autoxidation [11], activation of the polyol pathway [12], formation of advanced glycation end products (AGEs) [13], antioxidant enzyme inactivation [14] and an imbalance of glutathione redox status [15]. Hyperglycemia can promote an important oxidative imbalance, favoring the production of free radicals and the reduction of antioxidant defenses. At high concentrations, ROS/RNS can lead to damage to the major components of the cellular structure, including nucleic acids, proteins, amino acids, and lipids [16]. Such oxidative modifications in the diabetes condition would affect several cell functions, metabolism, and gene expression, which in turn can cause other pathological conditions [17].

It is important to note that the animal models of diabetes are very useful tools to gain new insights into human diabetes. Animal models induced by chemicals, such as alloxan, exhibit a syndrome of insulin resistance and type 2 diabetes [5]. Thus, the main objective of our study was to evaluate the effects of the animal model of diabetes induced by alloxan on the object recognition task and on the parameters of oxidative stress in the hippocampus, striatum, prefrontal cortex, and amygdala.

## 2. Material and Methods

### 2.1. Animals

Male Adult Wistar rats (60 days old) were obtained from the UNESC (Universidade do Extremo Sul Catarinense, Criciúma, SC, Brazil) breeding colony. They were housed five per cage with food and water available *ad libitum* and were maintained on a 12 h light/dark cycle (lights on at 7 : 00 a.m.). All experimental procedures involving animals were performed in accordance with the NIH Guide for the Care and Use of Laboratory Animals and the Brazilian Society for Neuroscience and Behavior (SBNeC) recommendations for animal care and with approval by the local Ethics Committee under protocol number 16/2010.

### 2.2. Diabetes Induction

Diabetes was induced in rats by using a single intraperitoneal injection of alloxan from Sigma Chemical Co. (St Louis, MO, USA) dissolved in a physiological saline (0.9% NaCl) solution (150 mg/kg), whereas the control group received only a saline injection [18]. Both groups were injected after an 18 h fasting period (60–70 mg/dL blood glucose). Fasting animals are more susceptible to alloxan probably due to partial protection by increased blood glucose [19]. All induced rats showed hyperglycemia (400–600 mg/dL) 48 h after alloxan administration. During the course of the experiment, the blood glucose level was monitored on a daily basis with commercial kits by performing a small puncture in the animals' tail. This methodology is quick and noninvasive, subjecting the rats to a negligible level of stress. At the end of the study, rats with glycemia between 400 and 600 mg/dL were considered diabetic [20]. Fifteen days after the induction of diabetes, based in previous studies [21], all rats were submitted to the object recognition task, after which the animals were killed by decapitation and a biochemical analysis was undertaken of the brain tissues.

### 2.3. Object Recognition Task

The object recognition task took place in a 40 × 60 cm open field surrounded by 50 cm high walls made of ply wood with a frontal glass wall. The floor of the open field was divided into 12 equal rectangles by black lines. All animals (alloxan or saline; *n* = 10–15 animals per group) were submitted to a habituation session where they were allowed to freely explore the open field for 5 min. No objects were placed in the box during the habituation trial. Twenty-four hours after habituation, training was conducted by placing the individual rats in the open field for 5 min, in which two identical objects (objects A1 and A2; both being cubes) were positioned in two adjacent corners, 10 cm from the walls. In a short-term recognition memory test given 1.5 h after training, the rats explored the open field for 5 min in the presence of one familiar (A) and one novel (B, a rectangle) object. In a long-term recognition memory test given 24 h after training, the rats explored the open field for 5 min in the presence of one familiar (A) and one novel (C, a pyramid with a square-shaped base) object. All objects had similar textures (smooth), colors (blue), and sizes (weight 150–200 g) but distinctive shapes. A recognition index calculated for each animal was calculated during the test session. It reports the ratio TB/(TA + TB) (TA = time spent exploring the familiar object A; TB = time spent exploring the novel object B) and it reports the ratio TC/(TA + TC) (TA = time spent exploring the familiar object A; TC = time spent exploring the novel object C). Between trials, the objects were washed with 10% ethanol solution. Exploration was defined as sniffing (exploring the object 3–5 cm away from it) or touching the object with the nose and/or forepaws [21].

### 2.4. Oxidative Stress Parameters

Immediately after the object recognition task, the animals were sacrificed by decapitation and the following brain areas; the prefrontal cortex, amygdala, hippocampus and striatum (*n* = 4–6 animals per group) were dissected according to the stereotaxic atlas [22] in ice-cold buffer, in a Petri dish. Submitochondrial particles were prepared in parallel from the four brain regions of each animal. For biochemical analysis in total tissue, the brain structures were rapidly frozen and stored at −70°C.

#### 2.4.1. Mitochondrial Isolation

Rat brain homogenates were centrifuged at 700 g for 10 min to discard nuclei and cell debris and the pellet was then washed to enrich the supernatant that was centrifuged at 700 g for 10 min. The obtained pellet, washed and resuspended in the same buffer, was considered to consist mainly of intact mitochondria able to carry out oxidative phosphorylation. The operations were carried out at 0–2°C. Submitochondrial particles (SMPs) were obtained by freezing and thawing (three cycles) of isolated mitochondria. For superoxide production measurements, SMP were washed twice with 140 mM KCl, 20 mM Tris-HCl (pH 7.4) and suspended in the same medium [23].

#### 2.4.2. Superoxide Production in Submitochondrial Particles of the Rat Brain

Superoxide production was determined in washed brain SMP using a spectrophotometric assay based on superoxide-dependent oxidation of epinephrine to adrenochrome at 37°C (€_480 nm_ = 4.0 mM^−1 ^cm^−1^). The reaction medium consisted of 0.23 M mannitol, 0.07 M sucrose, 20 mM Tris-HCl (pH 7.4), SMP (0.3–1.0 mg protein/mL), 0.1 *μ*M catalase, and 1 mM epinephrine. NADH (50 *μ*M) and succinate (7 mM) were used as substrates and rotenone (1 *μ*M) and antimycin (1 *μ*M) were added as specific inhibitors, respectively, to assay O_2_^−^ production at the NADH dehydrogenase and at the ubiquinone-cytochrome b region. Superoxide dismutase (SOD) was used at 0.1–0.3 *μ*M final concentration to give assay specificity [24].

#### 2.4.3. Thiobarbituric Acid Reactive Species Formation

To determine oxidative damage in lipid, we measured the formation of thiobarbituric acid reactive species (TBARS) during an acid-heating reaction, as previously described [25]. The samples were mixed with 1 mL of trichloroacetic acid 10% and 1 mL of thiobarbituric acid 0.67%, and then heated in a boiling water bath for 30 min. Malondialdehyde equivalents were determined in tissue and in submitochondrial particles of the rat brain spectrophotometrically by the absorbance at 532 nm.

#### 2.4.4. Carbonyl Protein Formation

Oxidative damage to proteins was assessed by the determination of carbonyl groups content based on the reaction with dinitrophenylhydrazine (DNPH), as previously described [26]. Proteins were precipitated by the addition of 20% trichloroacetic acid and were redissolved in DNPH. The absorbance was monitored spectrophotometrically at 370 nm.

#### 2.4.5. Superoxide Dismutase Activity

This method for the assay of superoxide dismutase (SOD) activity is based on the capacity of pyrogallol to autoxidize, a process highly dependent on O_2_^−2^; a substrate for SOD [27]. The inhibition of autoxidation of this compound thus occurs when SOD is present, and the enzymatic activity can be then indirectly assayed spectrophotometrically at 420 nm, using a double-beam spectrophotometer with temperature control. A calibration curve was performed using purified SOD as the standard, in order to calculate the specific activity of SOD present in the samples. A 50% inhibition of pyrogallol autoxidation is defined as 1 unit of SOD, and the specific activity is represented as units per mg of protein.

#### 2.4.6. Catalase Activity

The catalase (CAT) activity was assayed using a double-beam spectrophotometer with temperature control. This method is based on the disappearance of H_2_O_2_ at 240 nm in a reaction medium containing 20 mM H_2_O_2_, 0.1% Triton X-100, 10 mM potassium phosphate buffer, pH 7.0, and 0.1–0.3 mg protein/mL [28]. One CAT unit is defined as 1 mol of hydrogen peroxide consumed per minute, and the specific activity is reported as units per mg protein.

#### 2.4.7. Protein Determination

All biochemical measures were normalized to the protein content with bovine albumin as standard [29].

### 2.5. Statistical Analysis

In the open field test, the differences between training test sessions were analyzed by the paired Student's *t*-test. Data for recognition indexes are reported as median ± interquartile ranges (25 and 75). Comparisons among groups were performed using the Kruskal-Wallis test followed by Mann-Whitney test when necessary. The oxidative stress parameters were analyzed by Student's *t*-test for unpaired data and are reported as mean ± S.E.M. *P* values less than 0.05 were considered to be statistically significant.

## 3. Results

**Figure 1:**
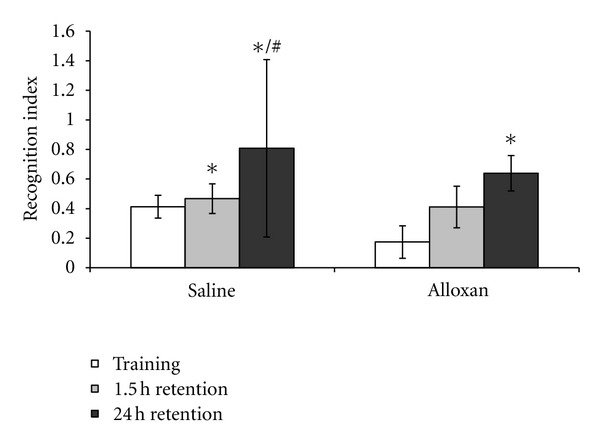
The effects of the animal model of diabetes induced by alloxan on the object recognition task. Results are reported as median ± interquartile ranges of the recognition indexes in training and short- and long-term memory retention test trials. *N* = 10–15 per group, *P < 0.05 difference from the training session and ^#^ *P* < 0.05 difference from the training from 1.5 h retention, according to Kruskal-Wallis test.

As depicted in Figure 1, in the object recognition task, no statistical differences were observed in the saline or alloxan groups in the training session (*P* > 0.05). In the control rats group, 1.5 h after the training session (short-term recognition memory), we observed an increase in the recognition index compared to the training session, and 24 h after the training session (long-term recognition memory) there was an increase in the recognition index, compared to the training session or to the short-term memory tests (*P* < 0.05; Figure 1). In the diabetic rats induced by alloxan, there was an increase in the recognition index after 24 h, but not 1.5 h after the training session (*P* < 0.05; Figure 1).

**Figure 2:**
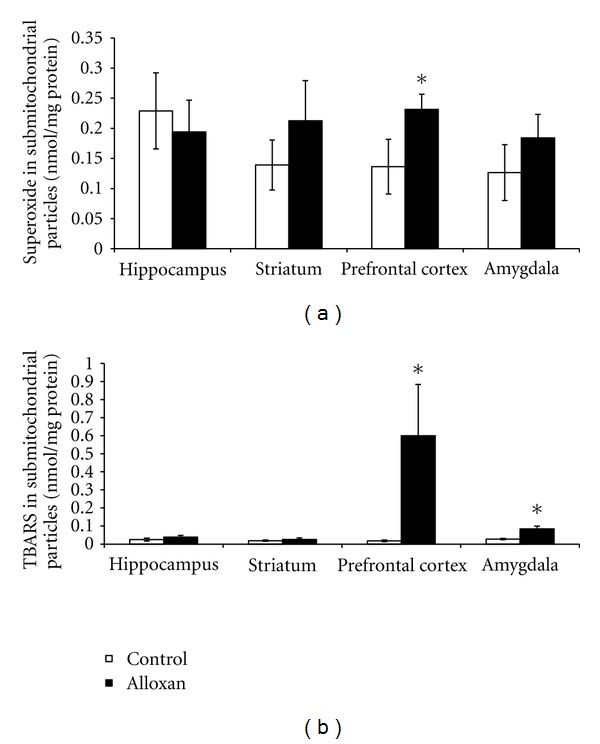
The effects of the animal model of diabetes induced by alloxan on the superoxide (a) and TBARS (b) production in submitochondrial particles in the hippocampus, striatum, prefrontal cortex and amygdala. Results are reported as mean + S.E.M. *N* = 4–6 per group, *P < 0.05 difference from the saline group, according to the Student *t*-test.

**Figure 3:**
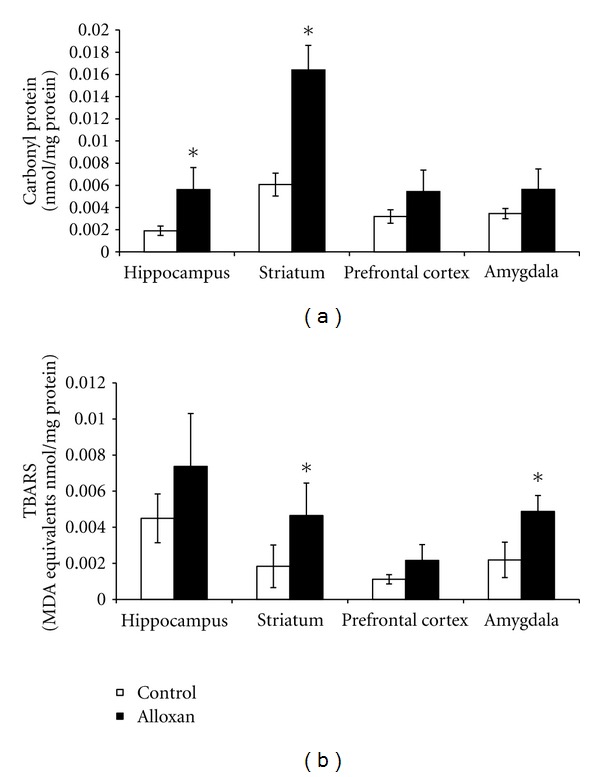
The effects of the animal model of diabetes induced by alloxan on TBARS production (a) and carbonyl formation (b) in the hippocampus, striatum, prefrontal cortex, and amygdala. Results are reported as mean + S.E.M. *N* = 4–6 per group, *P < 0.05 difference from the saline group, according to the Student's *t*-test.

**Figure 4:**
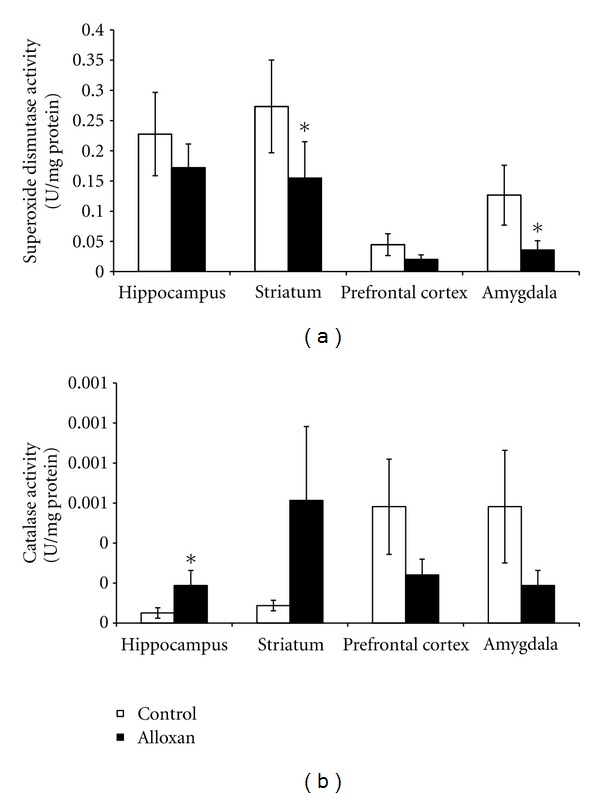
The effects of the animal model of diabetes induced by alloxan on the superoxide dismutase (a) and catalase (b) activities in the hippocampus, striatum, prefrontal cortex, and amygdala. Results are reported as mean + S.E.M. *N* = 4–6 per group, *P < 0.05 difference from the saline group, according to the Student's *t*-test.

In diabetic rats, there was an increase in the superoxide submitochondrial particles in the prefrontal cortex (*P* < 0.05; Figure 2(a)) and an increase in the TBARS submitochondrial particles in the prefrontal cortex and amygdala (Figure 2(b)). In diabetic rats it was shown that there was an increase in the carbonyl proteins in the hippocampus and striatum (*P* < 0.05; Figure 3(a)) and an increase in the TBARS oxidation in the striatum and amygdala (*P* < 0.05; Figure 3(b)). The SOD activity was decreased in diabetic rats in the striatum and amygdala (*P* < 0.05; Figure 4(a)). However, the CAT activity was increased in the hippocampus from diabetic rats (*P* < 0.05; Figure 4(b)).

## 4. Discussion

Experimental diabetes models can be induced by chemicals that selectively destroy the insulin-producing *β*-cells in the pancreas [30]. One of the most commonly used chemicals is alloxan. This drug induces diabetes by intracellular generation of ROS formed in a cyclic reaction involving alloxan and its reduced product called dialuric acid [18], with subsequent inhibition of insulin synthesis and secretion.

Recently, a significant body of evidence has accumulated to indicate that diabetes has detrimental effects on brain function. In fact, hypoglycemia and diabetes insults are related with damage in the hippocampus and hypothalamus, which are the brain areas associated with memory and plasticity functions [31]. A number of investigations have been performed to indicate that memory loss is a consequence of both type I and type II diabetes [3, 32]. However, the exact mechanism(s) as to how diabetic conditions could affect memory activity remains to be fully characterized. In the present study our results showed that in diabetic rats, there was an increase in the recognition index 24 h after the training session, indicating that diabetic rats did not alter recognition memory, when subjected to the object recognition task. Contrary to this, another study showed a significant reduction of memory formation, evaluated in the passive avoidance test in streptozotocin-induced diabetic mice [7]. The authors suggest that the overexpressed *β*-amyloid precursor might be one of the underlying factors causing memory deficit.

Differences between these studies could be related with the animal model of diabetes and tests used to evaluate memory. Although some studies have shown that oxidative stress induces significant deficits in cognitive performance (learning ability and memory retention) [33], in the present findings we did not show this correlation. It is important to note that during the formation of memories the activation of specific receptors and of several molecular cascades is required to convert extracellular signals that lead to changes in neuronal connectivity [34], which were probably not altered in the brains of alloxan-induced diabetic rats. For example, we recently demonstrated that brain-derived neurotrophic factor (BDNF) levels did not alter in the hippocampus from alloxan-induced diabetic rats [35]. BDNF is a neurotrophin which has an important role in hippocampal-dependent forms of memory [36]. Thus, the findings of the present study on recognition memory could be related, at least in part, because the animal model of diabetes induced by alloxan did not alter BDNF levels.

A previous study showed that alloxan-induced diabetes is associated with changes in the uptake of insulin by the brain, which includes increased binding to the capillary bed comprising the blood brain barrier and increased transport across the blood brain barrier [37]. Moreover, studies related that oxidative stress impacts several brain areas, such as the forebrain, cerebellum, and brain stem [31, 38]. Thus, in the present study we evaluated oxidative stress parameters in different brain areas, namely, the hippocampus, striatum, prefrontal cortex, and amygdala. Uncontrolled ROS production could lead to damage in cellular macromolecules (DNA, lipids, and protein) and other small antioxidant molecules [39], contributing to the progress of diabetic complications. Still, research indicates that obesity and hyperglycemia are associated with increases in the ROS production [40, 41]. In the present study we observed an increase in the superoxides in the prefrontal cortex and an increase in TBARS in the prefrontal cortex and amygdala in submitochondrial particles. Diabetes causes mitochondrial superoxide overproduction and this increased superoxide production is the major mediator of diabetes tissue damage [41]. In fact, in diabetic cells with a high intracellular glucose concentration, there is more glucose-derived pyruvate being oxidized in the tricarboxylic acid cycle, increasing the flux of electron donors (NADH and FADH2) into the electron transport chain [42]. Thus, electron transfer inside complex III is blocked [43], causing the electrons to use coenzyme Q as a backup, which donates the electrons one at a time to molecular oxygen, thereby generating superoxide [42]. The mitochondria are an organelle and have the ability to generate superoxides at complexes I and III [44, 45]. In addition to this, in brain tissue, complexes I and III have been attributed to major ROS production [44, 45]. In fact, a study from our group demonstrated that in alloxan-induced diabetic rats there were alterations in the mitochondrial respiratory chain [20]. Thus we suggest that alloxan-induced diabetic-like symptoms may provide a useful animal model to test the hypothesis of the involvement of oxidative stress in diabetes. Our results also showed an increase in carbonyl protein in the hippocampus, and amygdala and an increase in TBARS oxidation in the striatum and amygdala from diabetic animals. We discovered from our data that the TBARS levels in mitochondrial particles and in tissues were different, but our analysis was conducted in different areas, involving separate samples from submitochondrial particles and another from tissue. It is known that the mitochondria is a major producer of ROS [42], which in turn causes damage in lipids, which may explain the increase in TBARS in submitochondrial particles in the prefrontal cortex. Recently, Chang et al. [46] showed an increase in the carbonyl protein in the renal tissues from diabetic animals induced by streptozotocin. Additionally, similar to our findings, the TBARS index was increased in the liver of rats that had received a single injection of alloxan (150 mg/kg) [30]. Also, there was shown to be an increase in the TBARS in the plasma and hippocampus from diabetic animals induced by streptozotocin [47]. Recently, [48] investigated the effects of agmatine, an antihyperglycemic and antioxidant, on MDA and glutathione levels in the cerebral cortex and hippocampus from diabetic rats. The authors found that treatment with agmatine reduced oxidative stress markers in the brain of diabetic rats. In addition, [48] showed that brain regions differ in their response to oxidative stress in obese db/db mice. Still, silibinin, a compound with antioxidant properties, provided efficient neuroprotection in these diabetic rats [48]. Thus, this suggests that diabetes is related to oxidative stress in the brain.

 The present findings showed that SOD activity decreased in the striatum and amygdala. On the other hand, the CAT activity increased in the hippocampus in diabetic animals. SOD is a protective enzyme that can selectively scavenge the superoxide anion radical (O_2_^−.^) by catalyzing its dismutation to hydrogen peroxide (H_2_O_2_) [49]. CAT catalyzes degradation of H_2_O_2_ to water and O_2_. Another study showed that SOD and CAT activities were increased in the livers from alloxan-induced diabetic rats [31]. Also, Amer et al. [50] demonstrated that polymorphisms of glutathione S-transferase (an antioxidant enzyme) genes GSTM1 and GSTT1 were associated with an increased risk of type 2 DM. Interestingly, Di Naso et al. [51] reported that exogenous antioxidant copper zinc superoxide dismutase (Cu/Zn SOD) decreased liver peroxidation and increased nitric oxide synthase (NOS) in diabetic rats. Moreover, Gibson et al. [52] showed that N-acetylcysteine (NAC), a biosynthetic precursor of the antioxidant glutathione, reduced thrombotic propensity in type 2 diabetes patients, suggesting that this effect occurred by increasing the platelet antioxidant status as a result of elevated glutathione synthesis.

An imbalance in the SOD/CAT ratio indicates the generation of reactive species [53], which were reported in the present study. In this context, the effects of alloxan on SOD/CAT turnover (by increasing CAT and decreasing SOD activity) may result in antioxidant effects. An imbalance in the SOD/CAT ratio indicates the generation of reactive species, which were reported in the present study. Thus, considering the pathophysiology of diabetes, and the results presented in the present work, it is sensible to suggest that differences in oxidative stress parameters may be related, at least in part, with brain metabolism. Another study from our group also showed a decrease in SOD and an increase in CAT activities in the brain of rats submitted to the chronic mild stress procedure [54]. In fact, studies have reported a relationship between diabetes and stress [55, 56] inclusively, alloxan-induced diabetic rats presented depressive-like behaviour [20].

The cause of the overall oxidative imbalance demonstrated in our study may be due to mitochondrial dysfunction. Recently, it was shown that alloxan-induced diabetic rats presented alterations in the mitochondrial respiratory chain, creatine kinase, and citrate synthase activities [20]. However, mitochondrial alteration could occur by oxidative imbalance. In fact, ROS causes damage in the mitochondrial oxidative phosphorilation [56]. In addition, Bhattacharya et al. [57] reported decreased mitochondrial membrane potential, enhanced cytochrome c release, reciprocal regulation of the Bcl-2 family, and increases of caspases 3 and 9 in alloxan-induced diabetes. The authors also showed that treatment with D-saccharic acid 1,4-lactone, a derivative of D-glucaric acid which has antioxidant properties, counteracted these changes [57].

## 5. Conclusions

To our knowledge our data describes for the first time, the effects of the animal model of diabetes induced by alloxan on memory and oxidative stress parameters in the rat brain. In conclusion, alloxan-induced diabetes did not alter recognition memory, but induced oxidative damage and an imbalance between antioxidant enzymes, contributing, at least in part, to the pathophysiology of diabetes.
